# Analyzing Discrepancies in Chemical-Shift Predictions of Solid Pyridinium Fumarates

**DOI:** 10.3390/molecules26133857

**Published:** 2021-06-24

**Authors:** Martin Dračínský

**Affiliations:** Institute of Organic Chemistry and Biochemistry, Czech Academy of Sciences, Flemingovo nám. 2, 160 00 Prague, Czech Republic; martin.dracinsky@uochb.cas.cz

**Keywords:** solids, NMR spectroscopy, DFT calculations

## Abstract

Highly accurate chemical-shift predictions in molecular solids are behind the success and rapid development of NMR crystallography. However, unusually large errors of predicted hydrogen and carbon chemical shifts are sometimes reported. An understanding of these deviations is crucial for the reliability of NMR crystallography. Here, recently reported large deviations of predicted hydrogen and carbon chemical shifts of a series of solid pyridinium fumarates are thoroughly analyzed. The influence of the geometry optimization protocol and of the computational level of NMR calculations on the accuracy of predicted chemical shifts is investigated. Periodic calculations with GGA, meta-GGA and hybrid functionals are employed. Furthermore, molecular corrections at the coupled-cluster singles-and-doubles (CCSD) level are calculated. The effect of nuclear delocalization on the structure and NMR shielding is also investigated. The geometry optimization with a computationally demanding hybrid functional leads to a substantial improvement in proton chemical-shift predictions.

## 1. Introduction

In the past two decades, the progress of experimental and computational solid-state NMR (SS-NMR) methods has led to the rapid development of NMR crystallography, which combines theory and experiment to obtain otherwise inaccessible insights into the structure and dynamics of solids [1]. The success of NMR crystallography has been particularly driven by the possibility of fast and reliable computations of NMR parameters. A comparison of experimental and calculated chemical shifts and other NMR parameters with those calculated for a structural model is often used for the confirmation of the crystal structure, the discrimination between several different structural models or for de novo crystal-structure determination [2,3,4,5,6].

Most of the currently used computations of NMR parameters are based on density-functional theory (DFT) methods [7,8,9]. The most remarkable success has been achieved by the gauge-including projector-augmented wave (GIPAW) procedure, which was developed for the prediction of magnetic-resonance parameters in crystalline materials [10]. The GIPAW method has been implemented in several software packages that exploit translational periodicity in crystals. In these computations, periodic plane waves are used to form the basis set and the effective-core pseudopotentials are used to describe interactions close to the nuclei.

The computational level that can be used in combination with the GIPAW method has severe limitations. Plane-wave computations are usually performed with the general-gradient-approximation (GGA) family of density functionals, for example the Perdew–Burke–Ernzerhof (PBE) functional [11]. The GGA functionals are considered outdated for computations of non-periodic systems, but they are computationally very efficient for periodic systems. In comparison with the GGA functionals, the meta-GGA family of functionals adds the orbital kinetic energy density to the exchange-correlation functional. A meta-GGA functional, rSCAN, has recently been developed and implemented for condensed-matter simulations [12]. Electronic-structure calculations and geometry optimizations can also be performed with hybrid functionals, which incorporate a portion of exact exchange calculated at the Hartree–Fock level. These functionals are widely used for isolated molecules. Unfortunately, periodic plane-wave computations with hybrid functionals are almost two orders of magnitude more demanding on computational time and memory usage than those with GGA functionals. Nevertheless, several studies have demonstrated that going beyond the GGA level improves the accuracy of the predicted NMR parameters [13,14,15,16,17].

Despite the limitations of the computational level used in standard NMR crystallography studies, the predictions of carbon chemical shifts are usually surprisingly accurate with an estimated accuracy of approximately 2 ppm, which is usually sufficient for the purposes of NMR crystallography. However, several studies have reported much larger deviations. Corlett et al., for instance, have recently reported an NMR crystallography study of a series of pyridinium fumarates and observed differences of up to 6 ppm between the experimental and calculated carbon chemical shifts [18]. A similarly worrying discrepancy has been observed in GGA–GIPAW calculated carbon chemical shifts of solid testosterone, where most individual chemical shifts were reproduced to within a few ppm, with the notable exception of carbon C5, which was significantly overestimated [19,20].

We have recently investigated the factors contributing to the accuracy of the chemical-shift predictions of hydrogen nuclei in molecular solids and observed that the GGA-calculated proton chemical shifts deviated up to 1.5 ppm from the experiment, with the largest deviation observed for a hydrogen atom attached to sulfur, which has been explained by the neglect of relativistic effects in the calculations [21]. Furthermore, when hydrogen atoms are involved in strong hydrogen bonds, nuclear quantum effects (NQEs), such as proton delocalization and tunneling, may become important for the predictions of nuclear shielding [22,23,24,25]. However, with the exception of the hydrogen atom attached to the sulfur atom, the deviations from the experiment were always lower than 0.7 ppm, even with the standard GGA–GIPAW calculations. On the other hand, in the above-mentioned study of pyridinium fumarates, large deviations (up to 1.9 ppm) of the GIPAW predictions were observed [18].

These exceptions to the usually good accuracy of the predictions of SS-NMR parameters are worrying, because they undermine the credibility of the standard NMR crystallography approaches. Therefore, it is crucial to understand the reasons for these failures. Are they consequences of the choice of the DFT functional, of inherent inaccuracies in the DFT methodology or of inaccurate structural models?

**Figure 1:**
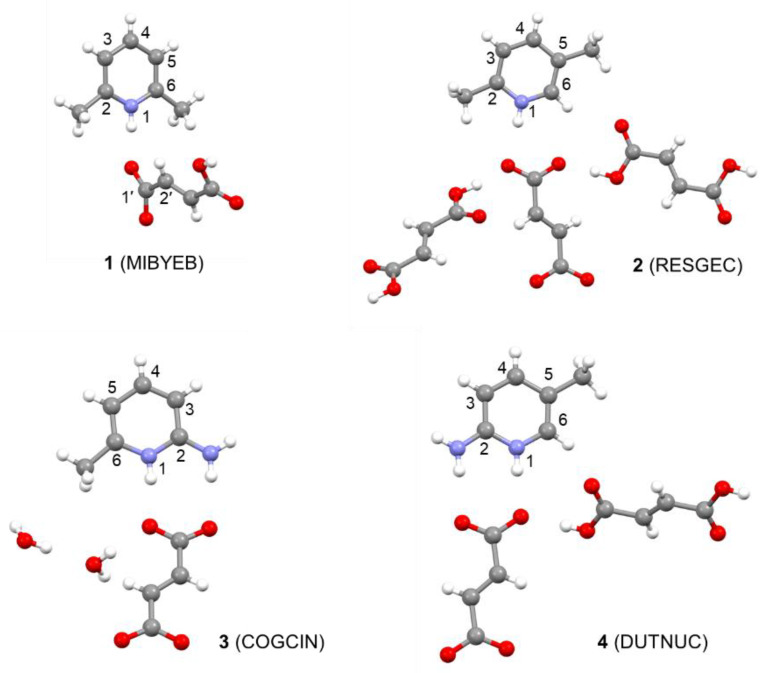
The structures of the systems studied together with their CSD ref codes (in parentheses) from the Cambridge Crystallographic Database [30] and atom numbering. Note that we employ the standard numbering of organic compounds and that the atom numbers differ from those used previously [18].

Here, we have selected the pyridinium fumarate systems reported by Corlett et al. [18] and we investigate the influence of the geometry optimization protocol and the computational level of NMR calculations on the accuracy of the predicted proton and carbon chemical shifts. We employ periodic calculations with GGA, meta-GGA and hybrid functionals. Furthermore, we apply molecular corrections at the coupled-cluster singles-and-doubles (CCSD) level, which serves as a benchmark for highly accurate quantum-chemical calculations. We also investigate the effect of nuclear delocalization on the structure and NMR shielding of a selected system. The structures of the systems studied, the pyridinium salts of fumaric acid, are shown in Figure 1. All the crystal structures have been determined using X-ray diffraction [26,27,28,29]. The crystal structures of some of the systems contain additional fumaric acid or water molecules.

## 2. Results

### 2.1. Geometry Optimization Protocol

The standard procedure that is most frequently used for the computations of NMR parameters with the GIPAW method includes the optimization of the positions of all atoms in the crystal structure obtained from an X-ray diffraction experiment or from a crystal-structure prediction tool. There are the following two reasons for the geometry optimization of experimental XRD structures: first, the characterization of the positions of hydrogen atoms is very challenging for XRD experiments and, second, molecular dynamics lead to the apparent shortening of the interatomic distances obtained by diffraction [31]. The geometry optimization is usually performed at the same computational level as the subsequent NMR calculation, i.e., with a GGA functional (typically the PBE functional) and an energy cutoff (the size of the basis set) of 500–700 eV. Nevertheless, it has been shown that the PBE functional tends to overestimate the covalent-bond distances of the hydrogen atoms involved in hydrogen bonding [32].

In addition to the standard level (PBE, 600 eV), we have also performed a geometry optimization of the studied systems with the computationally very demanding hybrid functional, B3LYP, and with the newly implemented meta-GGA functional, rSCAN. For the PBE and rSCAN levels, we have also tested the convergence with respect to the basis-set size. Table 1 summarizes the N1–H1 distances in the pyrimidium moiety optimized with the three functionals. These distances are, indeed, significantly longer in the PBE-optimized structures than in the structures optimized with the hybrid B3LYP functional. Note, however, that the geometry optimizations at the B3LYP level are impractical for routine computations because they are extremely demanding on computational resources. For example, the geometry optimization of the MIBYEB (**1**) system took approximately 12 days on 5 computational nodes, each with 36 cores. The distances calculated with the meta-GGA functional rSCAN are always between the PBE- and B3LYP-calculated distances, but they are closer to the B3LYP distances. The rSCAN functional may thus serve as a compromise providing geometries close to those obtained with a hybrid functional but with a small fraction of computational time (the computational time is approximately only 50% longer than that of the calculations with the PBE functional, about 3 h on 4 nodes for MIBYEB). The distances obtained with a larger energy cutoff of 900 eV (calculated only for the PBE and rSCAN functionals) are very close to the distances obtained with a cutoff of 600 eV (Tables S1–S3 in the SI). Other selected interatomic distances, including O–H, are also shown in the SI.

### 2.2. NMR Calculations—^1^H Chemical Shifts

As already pointed out in the original paper by Corlett et al. [18], there are significant deviations between the experimental proton chemical shifts and those calculated with the standard GIPAW procedure (PBE optimization, PBE calculation of NMR). In agreement with the previous report, we have observed the largest deviations for the fumaric-acid OH protons in the MIBYEB and RESGEC systems (deviations of 0.99 and 1.12 ppm, respectively). Table 2 summarizes the mean absolute errors (MAE) obtained and the maximum errors (E_max_) of the linear fit between the calculated shieldings and experimental shifts. The optimization with the larger basis set (an energy cutoff of 900 eV) has only negligible influence on the calculated MAE and E_max_ values (Table S4 in the SI).

The proton-shielding calculations (at the PBE level) performed on the geometries optimized at the B3LYP level are in substantially better agreement with the experiment than the calculations on the PBE-optimized geometries, with both the MAE and E_max_ values having dropped significantly. The largest maximum error (0.80 ppm) has been observed for the COGCIN system (Table 1).

The optimization at the rSCAN level and subsequent NMR calculation does not bring any clear advantage over the standard PBE method. Although the MAE and E_max_ values are slightly better than those obtained at the PBE level for the first two systems (MIBYEB and RESGEC), they are slightly worse for the remaining two systems (COGCIN and DUTNUC). The NMR calculations for the rSCAN-optimized structures were performed with both the PBE and rSCAN functionals with similar results. However, we have observed a significantly larger basis-set dependence for the rSCAN NMR calculations; increasing the cutoff energy to 900 eV improves the performance of the NMR predictions (see the SI).

### 2.3. NMR Calculations—^13^C Chemical Shifts

The correlation between the experimental ^13^C chemical shifts and shieldings calculated with the standard methodology (PBE optimization and NMR calculation) is reasonably good for the first two systems (MIBYEB and RESGEC). However, in agreement with the previous report [18], large discrepancies have been observed for carbon C2 in the COGCIN and DUTNUC systems (deviations of 4.6 and 5.9 ppm, respectively).

Geometry optimization at the B3LYP level does not improve the shielding–shift correlations. On the contrary, the MAE values are even larger for all the systems. Although the E_max_ values for the COGCIN and DUTNUC systems have dropped from 4.6 and 5.9 ppm to 3.3 and 4.0 ppm, respectively, the E_max_ values for the other two systems have increased from 1.9 and 2.7 ppm to 4.6 and 3.6 ppm, respectively.

The performance of the geometry optimization at the rSCAN level and the subsequent NMR calculation at the PBE level is comparable to that of the standard PBE/PBE procedure. However, the rSCAN calculations of the NMR parameters for structures optimized at the rSCAN level led to a significant improvement in the MAE values.

Interestingly, the largest error in the chemical-shift predictions obtained for the rSCAN- and B3LYP-optimized structures is not that of carbon C2, as in the case of the PBE calculations, but it is always one of the CH carbon atoms in the fumaric-acid residue.

The calculated nitrogen shieldings are reported in the SI; however, the experimental ^15^N chemical shifts for these structures have not been reported.

### 2.4. Salt/Cocrystal

We have also investigated the possibility that the experimental positioning of the acidic protons in the structures was incorrect. The distinction between salts and cocrystals depends on whether a proton transfer has occurred along the axis of a H-bond between the base and the acid, or not [33]. The distinction between these two types of crystalline forms is crucial for the pharmaceutical industry, not only because they often exhibit different physicochemical and pharmacokinetic performances, but they are also, from the legal and regulatory points of view, connected to intellectual property issues [34,35]. The structures of all the studied systems have been determined using X-ray diffraction experiments. However, hydrogen atoms (particularly those in short and strong H-bonds) are very difficult to characterize using this technique [36]. Furthermore, a previous computational study [37] concluded that DFT methods in several cases incorrectly placed the hydrogen atom on the base, i.e., they favored salts over cocrystals.

**Figure 2:**
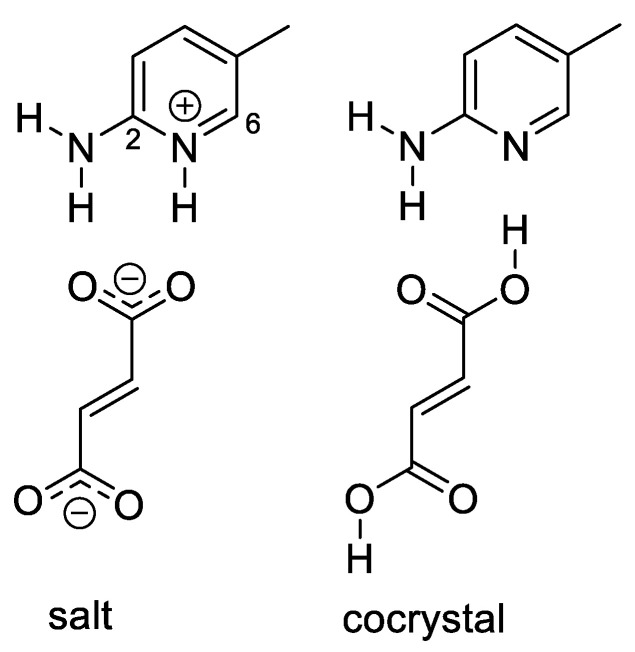
The salt and cocrystal forms of system **4** (DUTNUC).

We selected the DUTNUC system (**4**, Figure 2) for the investigation of the proton transfer between the acid and the base. All of the attempts to optimize the crystal structure in the cocrystal form failed; the optimization always led to the salt form. Nevertheless, when the position of the hydrogen atom was fixed on the fumaric-acid oxygen, the calculated shielding differed most significantly (with respect to the fully optimized salt form) for the carbon atoms C2 and C6 of the pyridine unit. The overall correlation of the experimental carbon chemical shifts with those calculated for the cocrystal form was significantly poorer than that of the salt form. This excludes the cocrystal form as the major structural pattern in the DUTNUC structure. However, partial delocalization of the hydrogen atom between the nitrogen and oxygen atoms cannot be excluded. The best agreement with the experiment was obtained when the shieldings were calculated as a weighted average of approximately 85% of the salt form and 15% of the cocrystal form (Table S7 in the SI).

### 2.5. Path-Integral Molecular Dynamics

We have further investigated the possibility of the hydrogen atom delocalization between the nitrogen and oxygen atoms in the hydrogen bond connecting the pyridine and fumarate fragments. An elegant and easy way to include nuclear quantum effects (NQEs) in quantum-chemical simulations is based on the path-integral [38] (PI) formalism. We have employed a path-integral molecular dynamics (PIMD) simulation, which was shown previously to be an excellent tool for the investigation of NQEs, such as hydrogen atom delocalization and tunneling [22,23,24,25].

**Figure 3:**
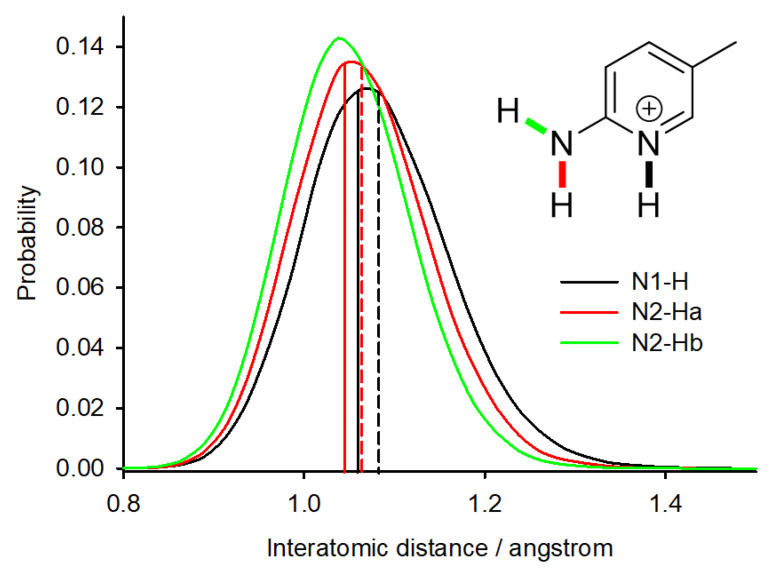
The probability distribution of N–H interatomic distances in system **4** (DUTNUC) obtained from the PIMD simulation. The solid lines indicate the N–H distance in the geometry-optimized structure and the dashed lines indicate the average N–H distance observed during the PIMD simulation.

We have performed the PIMD simulation for system **4** (DUTNUC) and analyzed the delocalization of the acidic hydrogens by plotting the probabilities of selected interatomic distances (Figure 3). The average N–H distance of the pyridine nitrogen (N1) is significantly larger (1.083 Å) than the distance in the structure optimized at the same computational level (1.059 Å). Furthermore, the delocalization of this proton is larger (the probability distribution is broader) than the delocalization of the other two N–H protons present in the molecule. The PIMD simulation thus supports the above-discussed possible partial presence of the cocrystal form in the crystal structure of DUTNUC.

### 2.6. CCSD Corrections

We have previously proposed a methodology for a simple correction to the shielding values of molecular crystals calculated at the PBE level [20]. Briefly, the crystal structure is optimized, and the NMR parameters are calculated at the PBE level with periodic conditions. Subsequently, the shielding values at the PBE level and at a higher computational level are calculated for a single molecule extracted from the optimized crystal structure, and the difference between these two single-molecule calculations is added to the shielding values calculated with the periodic conditions as a correction. This methodology was particularly successful for the calculations of carbon chemical shifts; the correlation between predictions and experiment improved significantly [20].

Here, we have applied this methodology and calculated the single-molecule corrections at the currently highest possible computational level, coupled-cluster singles and doubles (CCSD), for the two systems with large errors in the calculated carbon chemical shifts (COGCIN and DUTNUC). Note that the CCSD computations are extremely demanding, and the size of the investigated systems is probably at the limit of what can currently be calculated.

When the molecular corrections calculated at the CCSD level are added to the carbon shieldings calculated at the PBE level, the correlation with the experiment is significantly better (Table 2).

## 3. Conclusions

The rapid development of NMR crystallography in the past two decades is largely a result of the availability of fast and reliable computational predictions of the NMR chemical shifts of solids. The GIPAW method is probably the most successful method for the chemical-shift calculations of crystalline solids. This methodology has been particularly successful and the most documented for the calculation of carbon chemical shifts. Very good agreement between the calculations and the experiment is usually observed. This success of the GIPAW methodology is somewhat surprising given the many approximations used in the calculations. The most critical limitation is probably that only GGA functionals (most often the PBE functional) can be widely used with the GIPAW method. Al-though the computations are very successful, several worrying cases have been reported with large deviations of the calculated shifts from the experiment. As these deviations undermine the reliability of the NMR crystallography methodology, it is crucial to understand the reasons for the failures.

In this paper, we have investigated the large discrepancies previously observed between the experimental and calculated carbon and hydrogen chemical shifts in the fumarate salts of pyridine derivatives. We have employed the newly implemented meta-GGA functional rSCAN and the computationally very demanding hybrid functional B3LYP for geometry optimizations. The bond distances between the atoms involved in intermolecular H-bonds are significantly shorter in the structures optimized at the B3LYP level than in the structures optimized at the standard PBE level. The bond distances in the rSCAN-optimized structures are closer to the B3LYP-derived distances, and the geometry optimization at the rSCAN level may thus serve as a fast and more accurate alternative to the standard PBE level.

The inaccurate distances obtained at the standard PBE level of approximation may be the reason for the larger discrepancies between the experimental and predicted proton chemical shifts. Indeed, the proton chemical shifts calculated for the B3LYP geometries are in very good agreement with the experiment. However, the geometry optimization of periodic systems at the B3LYP level is extremely computationally demanding and cannot be used routinely. The performance of the rSCAN functional for NMR calculations is not convincing—we have observed only a modest improvement in the predictions of proton chemical shifts. Further investigation of the performance of this functional for NMR calculations on a larger set of systems is necessary.

The geometry optimization at the expensive B3LYP level had an ambiguous effect on the performance of carbon chemical-shift predictions. The largest deviations observed for the PBE predictions for carbon C2 in the pyridinium moiety decreased; at same time, however, the B3LYP geometry optimization deteriorated the very good predictions of carbon chemical shifts of the first two systems (MIBYEB and RESGEC).

We have also investigated the effect of the partial delocalization of the hydrogen atom in the intermolecular hydrogen bond between the fumarate and pyridinium moieties of system **4** (DUTNUC). NMR calculations for the structure with the hydrogen atom fixed on the fumarate oxygen (i.e., a cocrystal instead of a salt) have revealed that the position of the hydrogen atom most significantly affects the chemical shifts of the carbon atoms C2 and C6. A partial presence of the cocrystal form (about 15%) leads to a significant drop in the deviations of the predicted carbon chemical shifts. Partial delocalization of the hydrogen atom between the acid and the base has also been observed in the PIMD simulations.

Additionally, we have calculated single-molecule corrections to the predicted carbon shieldings at the highly accurate CCSD level. These corrections have also improved the agreement between the calculated and experimental carbon chemical shifts of the two most problematic systems (COGCIN and DUTNUC).

It may seem paradoxical that the geometry optimization at the B3LYP level leads to shorter bond distances and better predictions of hydrogen chemical shifts and, on the other hand, the PIMD simulation reveals that the average bond distances are longer than in the geometry-optimized structures and, taking the hydrogen atom delocalization into account, improves the carbon chemical shifts. We hypothesize that the geometry optimizations using the standard PBE functional may lead to a fortunate error cancellation and a good estimation of the finite temperature bond distances and accurate predictions of the carbon chemical shifts in most cases. However, in the “problematic” systems, such as those with short strong H-bonds, this error cancellation is incomplete, and the chemical-shift predictions are less accurate.

In summary, we can conclude that the geometry optimization level is crucial for the predictions of hydrogen atom positions and proton chemical shifts. Systems with strong intermolecular hydrogen bonds may be particularly sensitive to the geometry optimization level. Furthermore, NMR calculations for geometry-optimized structures do not include the possible effect of hydrogen atom delocalization, such as the partial presence of both the salt and cocrystal forms.

## 4. Methods

### 4.1. Structures

The structures of the studied systems determined using X-ray diffraction (CSD ref codes MIBYEB, RESGEC, COGCIN and DUTNUC) were obtained from the Cambridge Crystallographic Database [30].

### 4.2. Geometry Optimization

The positions of all atoms with fixed unit-cell parameters were optimized using the CASTEP program [39], version 20.11, which is a DFT-based code that uses pseudopotentials to model the effects of core electrons and plane waves to describe the valence electrons. Electron-correlation effects were modeled using the GGA functional PBE [11], the meta-GGA functional rSCAN [12], or the hybrid functional B3LYP [40,41]. The optimization was performed utilizing the plane-wave basis-set energy cutoff of 600 or 900 eV, default ‘on-the-fly generation’ pseudopotentials for PBE and rSCAN calculations and norm-conserving pseudopotentials for B3LYP calculations, and a minimum k-point spacing of 0.05 Å^−1^ over the Brillouin zone via a Monkhorst–Pack grid [42]. Empirical dispersion corrections TS [43] were used for the PBE and B3LYP calculations, and MBD [44] for the rSCAN calculations.

### 4.3. NMR Shieldings in Infinite Crystals

The NMR shieldings of the geometry-optimized structures were calculated at the PBE or rSCAN level using the GIPAW approach [10,45]. The shielding values of the three equivalent hydrogen atoms in methyl groups were averaged before the comparison with experimental values.

### 4.4. PIMD Simulations

The PIMD simulation of system 4 (DUTNUC) was also performed in CASTEP using an NVT ensemble, a temperature of 300 K, a Langevin thermostat, a 0.5-fs integration time step, ultrasoft pseudopotentials [46] and a planewave cutoff energy of 300 eV. Integrals were taken over the Brillouin zone using a Monkhorst–Pack [42] grid of the minimum k-point sampling of 0.1 Å^–1^. Electron-correlation effects were modeled using the PBE functional. The atomic positions were optimized by energy minimization prior to the MD runs at the same computational level. The lattice parameters were fixed to the experimental values. No symmetry constraints were applied during the runs, as these are only relevant to the time-averaged structure. After 1 ps of equilibration, a 5-ps-long productive simulation run was performed. The path-integral propagation used a Trotter decomposition of all nuclei into 16 beads, which has been shown to be sufficient for simulations of molecular crystals at 300 K [23].

### 4.5. Isolated-Molecule Corrections

DFT NMR shieldings for the isolated molecules (in vacuum) were calculated using the Gaussian16 program [47]. The gas-phase molecule-input geometries were taken from the periodic DFT geometry-optimized structures and were not further optimized. The PBE functional together with the 6-31+G(d,p) basis set were used for the calculations. NMR shieldings at the CCSD [48,49,50,51] level and the 6-31+G(d,p) basis set were calculated with the CFOUR program package, which is suitable for performing high-level quantum-chemical calculations on atoms and molecules [52,53]. The CCSD correction was obtained as the difference between the CCSD and PBE chemical shieldings.

## Figures and Tables

**Table 1:** The N1–H1 distances (Å) in the pyrimidium species obtained after the geometry optimization of the crystal structures with the PBE, B3LYP and rSCAN functionals and an energy cutoff of 600 eV.

	PBE	B3LYP	rSCAN
MIBYEB	1.086	1.063	1.070
RESGEC	1.058	1.042	1.047
COGCIN	1.065	1.047	1.051
DUTNUC	1.055	1.039	1.044

**Table 2:** The mean absolute errors (MAE) and the maximum errors E_max_ (ppm) of the linear fit between experimental chemical shifts and calculated shieldings (a cutoff energy of 600 eV).

Optimization		PBE		B3LYP		rSCAN		rSCAN		PBE	
NMR Calculation		PBE		PBE		PBE		rSCAN		PBE+CCSD *^a^*	
		MAE	E_max_	MAE	E_max_	MAE	E_max_	MAE	E_max_	MAE	E_max_
^1^H	MIBYEB	0.29	0.99	0.13	0.39	0.26	0.98	0.28	0.93	–	–
	RESGEC	0.33	1.12	0.21	0.66	0.28	0.96	0.24	0.81	–	–
	COGCIN	0.18	0.50	0.23	0.80	0.24	0.80	0.24	0.82	0.14	0.46
	DUTNUC	0.27	0.56	0.20	0.59	0.36	0.78	0.35	0.86	0.32	0.72
	all	0.46	1.17	0.38	0.81	0.50	1.21	0.47	1.16	–	–
^13^C	MIBYEB	1.04	1.93	1.31	4.64	1.09	3.81	0.98	3.41	–	–
	RESGEC	1.24	2.65	1.93	3.56	1.61	3.65	1.25	2.93	–	–
	COGCIN	1.56	4.60	1.81	3.31	1.58	4.55	1.21	3.11	1.65	2.90
	DUTNUC	1.78	5.85	1.86	4.01	1.76	5.53	1.38	4.16	2.00	3.30
	all	1.87	6.41	2.20	5.11	2.06	6.11	1.63	4.53	–	–

*^a^* Periodic calculation at the PBE level with isolated-molecule corrections at the CCSD level.

## Supplementary Materials

The following are available online, Table S1: The C4–H4 distances (Å) in the pyrimidium species obtained after geometry optimization of the crystal structures with the PBE, B3LYP and rSCAN functionals and energy cutoffs of 600 and 900 eV, Table S2: The O–H distances (Å) in the pyrimidium species obtained after geometry optimization of the crystal structures with the PBE, B3LYP and rSCAN functionals and energy cutoffs of 600 and 900 eV, Table S3: The N1–H1 distances (Å) in the pyrimidium species obtained after geometry optimization of the crystal structures with the PBE and rSCAN functionals and energy cutoffs of 600 and 900 eV, Table S4: Mean absolute errors and maximal errors (ppm) of the linear fit between experimental chemical shifts and calculated shieldings (PBE or rSCAN functional, cutoff energy of 600 or 900 eV), Tables S5–S8: Experimental chemical shifts ([18] in the main text) and calculated shieldings (ppm) of MIBYEB, RESGEC, COGCIN and UDTNUC. The atom numbering corresponds to the numbering in the crystal structure deposited in CSD. Parameters of the linear fit between the experimental shifts and calculated shieldings, Table S10: The calculated shieldings (*σ*) and chemical shifts (*δ*) of the N–H and O–H protons in DUTNUC and the corresponding bond distances (Å). All NMR calculations were performed with the PBE functional and *E*_cut_ = 600 eV.

## Sample Availability

Not available.
