# Supplementary material for: Analyzing Discrepancies in Chemical-Shift Predictions of Solid Pyridinium Fumarates
Source: Molecules. 2021 Jun 24;26(13):3857. doi: 10.3390/molecules26133857 (PMC8270278; doi:10.3390/molecules26133857)
Supplement: Supplementary file 1 [file molecules-26-03857-s001.zip › molecules-1274213-supplementary.pdf]

Supplementary Materials

Analyzing Discrepancies in Chemical-Shift Predictions of Solid Pyridinium Fumarates

Martin Dračínský

Institute of Organic Chemistry and Biochemistry, Czech Academy of Sciences, Flemingovo nám. 2, 160 00 Prague 6, Czech Republic; martin.dracinsky@uochb.cas.cz

Table S1. The C4–H4 distances (Å) in the pyrimidium species obtained after geometry optimization of the crystal structures with the PBE, B3LYP and rSCAN functionals and energy cutoffs of 600 and 900 eV.

**Functional**	**PBE**	**PBE**	**B3LYP**	**rSCAN**	**rSCAN**
E_{cut} / eV	600	900	600	600	900
MIBYEB	1.088		1.072	1.080	1.080
RESGEC	1.092	1.091	1.071	1.083	1.083
COGCIN	1.089	1.088	1.073	1.081	1.080
DUTNUC	1.090	1.090	1.074	1.083	1.082

Table S2. The O–H distances (Å) in the pyrimidium species obtained after geometry optimization of the crystal structures with the PBE, B3LYP and rSCAN functionals and energy cutoffs of 600 and 900 eV.

**Functional**	**PBE**	**PBE**	**B3LYP**	**rSCAN**	**rSCAN**
E_{cut} / eV	600	900	600	600	900
MIBYEB	1.053		1.018	1.035	1.032
RESGEC	1.031, 1.033	1.031, 1.033	1.006, 1.007	1.016, 1.017	1.014, 1.016
COGCIN	–	–	–	–	–
DUTNUC	1.034	1.035	1.011	1.021	1.020

Table S3. The N1–H1 distances (Å) in the pyrimidium species obtained after geometry optimization of the crystal structures with the PBE and rSCAN functionals and energy cutoffs of 600 and 900 eV.

**Functional**	**PBE**	**PBE**	**rSCAN**	**rSCAN**
E_{cut} / eV	600	900	600	900
MIBYEB	1.086		1.070	1.072
RESGEC	1.058	1.059	1.047	1.046
COGCIN	1.065	1.065	1.051	1.053
DUTNUC	1.055	1.055	1.044	1.043

Table S4. Mean absolute errors and maximal errors (ppm) of the linear fit between experimental chemical shifts and calculated shieldings (PBE or rSCAN functional, cutoff energy of 600 or 900 eV).

**Optimization**		**PBE600**		**PBE900**		**PBE900**		**rSCAN600**		**rSCAN900**		**rSCAN900**	
**NMR calc.**		**PBE600**		**PBE600**		**PBE900**		**rSCAN600**		**rSCAN600**		**rSCAN900**	
		MAE	E_{max}	MAE	E_{max}	MAE	E_{max}	MAE	E_{max}	MAE	E_{max}	MAE	E_{max}
^{1}H	MIBYEB	0.29	0.99	0.30	1.01	0.31	1.04	0.28	0.93	0.22	0.57	0.23	0.62
	RESGEC	0.33	1.12	0.34	1.06	0.34	1.06	0.24	0.81	0.25	0.83	0.25	0.83
	COGCIN	0.18	0.50	0.19	0.58	0.19	0.59	0.24	0.82	0.21	0.78	0.21	0.77
	DUTNUC	0.27	0.56	0.28	0.56	0.28	0.56	0.35	0.86	0.30	0.61	0.30	0.62
^{13}C	MIBYEB	1.04	1.93	1.06	2.10	1.02	1.96	0.98	3.41	0.85	3.56	0.91	3.49
	RESGEC	1.24	2.65	1.35	2.93	1.28	2.81	1.25	2.93	1.38	3.01	1.34	2.86
	COGCIN	1.56	4.60	1.51	4.50	1.54	4.62	1.21	3.11	1.13	2.83	1.21	3.04
	DUTNUC	1.78	5.85	1.81	5.69	1.77	5.85	1.38	4.16	1.47	4.30	1.45	4.47

Table S5. Experimental chemical shifts (ref. [18] in the main text) and calculated shieldings (ppm) of MIBYEB. The atom numbering corresponds to the numbering in the crystal structure deposited in CSD. Parameters of the linear fit between the experimental shifts and calculated shieldings.

**Opt-funct.**		PBE	PBE	PBE	B3LYP	rSCAN	rSCAN	rSCAN	rSCAN
**Opt-E_{cut}**		600	900	900	600	600	600	900	900
NMR-funct.		PBE	PBE	PBE	PBE	rSCAN	PBE	rSCAN	rSCAN
NMR-E_{cut}		600	600	900	600	600	600	900	600
Atom	Exp								
H13	15.8	12.93	12.90	13.03	14.71	13.30	13.54	13.65	13.55
H11	7.9	22.43	22.46	22.64	22.91	22.19	22.66	22.36	22.18
H12	7.5	22.91	22.96	23.15	23.62	22.86	23.28	23.08	22.90
H10	17.7	12.66	12.63	12.81	13.51	12.93	13.30	12.65	12.49
H4		26.81	26.80	26.97	27.23	26.27	26.83	26.61	26.44
H5		28.88	28.90	29.08	29.41	28.62	29.17	28.75	28.57
H6		29.33	29.31	29.49	29.80	29.04	29.58	29.21	29.02
H1	6.3	24.14	24.14	24.33	24.56	23.96	24.36	24.16	23.98
H2	6.9	23.62	23.63	23.82	23.95	23.38	23.81	23.54	23.35
H3	7.9	22.53	22.56	22.76	23.07	22.51	22.84	22.71	22.51
H7		28.80	28.80	28.99	29.27	28.46	29.05	28.65	28.46
H8		28.77	28.77	28.95	29.17	28.43	28.98	28.57	28.40
H9		27.42	27.41	27.59	27.97	27.07	27.61	27.37	27.18
	MAE	0.29	0.30	0.31	0.13	0.28	0.26	0.23	0.22
	E_{max}	0.99	1.01	1.04	0.39	0.93	0.98	0.62	0.57
	Slope	-1.060	-1.062	-1.064	-1.001	-1.014	-1.029	-1.029	-1.026
	Intercept	30.725	30.751	30.946	30.910	30.258	30.802	30.545	30.342
C8	173.4	-4.79	-4.16	-5.12	3.65	9.83	-1.35	9.98	10.76
C9	142.7	24.51	24.68	24.56	26.69	36.25	24.80	36.09	36.39
C10	132.4	35.13	35.12	34.99	41.84	49.29	38.43	49.53	49.84
C11	169.9	-2.15	-1.46	-2.27	5.50	12.12	1.32	12.19	12.91
C6	19.6	154.27	154.23	154.23	157.90	159.33	155.83	159.35	159.45
C1	152.6	17.26	17.23	17.09	20.88	30.61	19.36	30.56	30.77
C2	123.8	45.57	45.56	45.46	50.08	58.62	47.79	58.46	58.59
C3	146.4	24.22	24.35	24.26	27.96	37.70	26.03	37.28	37.55
C4	128.2	40.52	40.51	40.40	45.60	54.18	43.01	53.88	54.10
C5	152.6	17.51	17.59	17.45	20.72	30.79	19.29	30.60	30.86
C7	19.6	155.52	155.47	155.45	158.96	160.74	157.05	160.85	160.92
	MAE	1.04	1.06	1.02	1.31	0.98	1.09	0.91	0.85
	E_{max}	1.93	2.10	1.96	4.64	3.41	3.81	3.49	3.56
	Slope	-1.041	-1.038	-1.041	-1.025	-0.979	-1.033	-0.979	-0.976
	Intercept	174.920	174.718	174.895	177.784	179.232	176.213	179.262	179.212
N		15.09	15.28	13.70	22.46	35.53	20.89	32.92	34.29

Table S6. Experimental chemical shifts (ref. [18] in the main text) and calculated shieldings (ppm) of RESGEC. The atom numbering corresponds to the numbering in the crystal structure deposited in CSD. Parameters of the linear fit between the experimental shifts and calculated shieldings.

**Opt-funct.**		PBE	PBE	PBE	B3LYP	rSCAN	rSCAN	rSCAN	rSCAN
**Opt-E_{cut}**		600	900	900	600	600	600	900	900
NMR-funct.		PBE	PBE	PBE	PBE	rSCAN	PBE	rSCAN	rSCAN
NMR-E_{cut}		600	600	900	600	600	600	900	600
Atom	Exp								
H10	14.3	16.06	15.91	16.05	16.58	15.74	16.23	15.91	15.77
H1	6.4	23.08	23.06	23.24	23.51	22.99	23.31	23.12	22.94
H2	7.1	22.78	22.81	22.98	23.22	22.67	23.06	22.84	22.67
H3	8.1	21.65	21.62	21.80	22.24	21.43	21.86	21.66	21.47
H4		28.71	28.67	28.86	29.23	28.43	28.95	28.63	28.44
H5	2.1	28.47	28.45	28.64	28.91	28.13	28.69	28.29	28.11
H6		27.43	27.53	27.71	28.03	27.44	27.94	27.63	27.44
H7		27.85	27.81	27.99	28.44	27.69	28.14	27.90	27.70
H8	2.1	28.61	28.60	28.78	29.10	28.39	28.89	28.56	28.37
H9		28.21	28.18	28.36	28.71	27.96	28.47	28.19	28.00
H13	6.4	23.64	23.61	23.79	24.06	23.42	23.83	23.58	23.41
H12	13.4	15.29	15.14	15.26	16.83	15.52	15.78	15.93	15.80
H11	5.6	24.13	24.10	24.26	24.58	23.79	24.27	23.92	23.76
H14	13.4	14.86	14.85	14.97	16.25	15.15	15.40	15.15	15.02
H15	6.4	23.49	23.47	23.64	23.90	23.17	23.60	23.37	23.19
	MAE	0.33	0.34	0.34	0.21	0.24	0.28	0.25	0.25
	E_{max}	1.12	1.06	1.06	0.66	0.81	0.96	0.83	0.83
	Slope	-1.074	-1.083	-1.088	-1.013	-1.048	-1.061	-1.047	-1.042
	Intercept	30.213	30.244	30.445	30.407	29.879	30.385	30.049	29.847
C1	152.4	17.08	17.11	16.94	20.29	30.10	19.03	29.77	30.14
C2	128.1	42.79	42.50	42.38	47.22	55.72	44.80	55.39	55.56
C3	146.9	23.97	23.98	23.87	27.70	37.33	25.82	36.93	37.21
C4	136.0	32.65	32.56	32.42	37.53	46.44	34.85	46.44	46.67
C5	142.5	28.34	28.38	28.19	32.92	41.86	31.00	42.10	42.33
C6	16.8	156.95	157.16	157.13	160.26	162.41	158.99	162.50	162.61
C7	16.8	157.57	157.53	157.5	161.02	163.08	159.49	163.01	163.14
C11	136.0	31.54	31.38	31.26	35.23	44.35	33.25	43.92	44.23
C10	169.8	-0.77	-0.07	-0.86	7.09	14.08	3.05	13.90	14.65
C8	173.5	-3.99	-3.14	-4.02	3.68	11.26	0.09	10.78	11.56
C9	136.0	30.77	30.79	30.66	34.82	43.91	32.83	43.29	43.56
C12	168.1	-0.09	0.49	-0.29	7.80	14.82	4.03	14.15	14.93
C13	136.0	30.68	30.55	30.35	34.88	43.50	32.20	43.41	43.53
	MAE	1.24	1.35	1.28	1.93	1.25	1.61	1.34	1.38
	E_{max}	2.65	2.93	2.81	3.56	2.93	3.65	2.86	3.01
	Slope	-1.035	-1.033	-1.037	-1.016	-0.974	-1.027	-0.976	-0.973
	Intercept	174.224	174.068	174.240	176.572	178.780	175.614	178.856	178.807
N		24.80	24.56	23.01	30.40	42.60	28.67	41.78	43.06

Table S7. Experimental chemical shifts (ref. [18] in the main text) and calculated shieldings (ppm) of COGCIN. The atom numbering corresponds to the numbering in the crystal structure deposited in CSD. Parameters of the linear fit between the experimental shifts and calculated shieldings.

**Opt-funct.**		PBE	PBE	PBE	B3LYP	rSCAN	rSCAN	rSCAN	rSCAN
**Opt-E_{cut}**		600	900	900	600	600	600	900	900
NMR-funct.		PBE	PBE	PBE	PBE	rSCAN	PBE	rSCAN	rSCAN
NMR-E_{cut}		600	600	900	600	600	600	900	600
Atom	Exp								
H1	6.0	23.57	23.60	23.77	24.08	23.41	23.82	23.60	23.45
H_2O		24.24	24.28	24.38	25.01	24.40	24.80	24.50	24.41
H_2O		23.84	23.77	23.85	24.60	24.47	24.82	24.33	24.24
H_2O		25.07	25.18	25.29	25.54	24.83	25.20	25.79	25.70
H_2O		25.45	25.41	25.51	26.05	26.08	26.45	25.77	25.68
H14	8.2	21.29	21.20	21.37	21.87	21.06	21.55	21.39	21.26
H15	8.2	22.14	22.20	22.38	23.02	22.34	22.77	22.43	22.29
H13	14.9	14.90	14.83	14.99	15.63	14.95	15.48	14.74	14.65
H16	6.0	23.77	23.74	23.93	24.27	23.58	24.00	23.78	23.61
H21	6.8	23.16	23.13	23.32	23.61	22.93	23.35	23.12	22.96
H20	5.3	24.58	24.55	24.73	24.99	24.43	24.81	24.59	24.44
H17		28.08	28.07	28.26	28.56	27.80	28.31	27.95	27.79
H18	1.5	29.30	29.29	29.48	29.78	29.02	29.55	29.18	29.01
H19		27.91	27.92	28.10	28.36	27.51	28.10	27.79	27.63
	MAE	0.18	0.19	0.19	0.23	0.24	0.24	0.21	0.21
	E_{max}	0.50	0.58	0.59	0.80	0.82	0.80	0.77	0.78
	Slope	-1.004	-1.009	-1.011	-0.978	-0.975	-0.972	-1.006	-1.000
	Intercept	29.870	29.884	30.077	30.250	29.537	29.966	29.897	29.710
C1	174.4	-5.81	-5.07	-5.95	2.17	9.64	-1.85	8.86	9.87
C2	137.6	30.82	30.83	30.74	34.35	43.41	32.06	43.49	43.78
C9	155.5	19.53	19.51	19.35	22.78	31.34	21.76	30.94	31.17
C13	110.4	60.39	60.51	60.42	64.44	71.98	62.52	72.05	72.17
C14	145.4	26.18	26.01	25.91	30.50	39.18	28.02	38.93	39.27
C11	110.4	59.59	59.51	59.40	63.48	71.13	61.43	71.11	71.44
C10	149.2	20.22	19.96	19.82	23.47	33.06	22.00	32.62	32.92
C12	20.9	152.68	152.81	152.79	156.34	158.24	154.46	158.25	158.41
	MAE	1.56	1.51	1.54	1.81	1.21	1.58	1.21	1.13
	E_{max}	4.60	4.50	4.62	3.31	3.11	4.55	3.04	2.83
	Slope	-1.019	-1.019	-1.022	-1.007	-0.963	-1.012	-0.967	-0.964
	Intercept	173.353	173.316	173.508	176.072	178.026	174.589	178.337	178.286
N1		141.92	141.98	140.79	147.72	153.70	145.79	153.44	154.42
N2		60.99	60.82	59.42	67.06	77.52	66.15	74.76	75.95

Table S8. Experimental chemical shifts (ref. [18] in the main text) and calculated shieldings (ppm) of DUTNUC. The atom numbering corresponds to the numbering in the crystal structure deposited in CSD. Parameters of the linear fit between the experimental shifts and calculated shieldings.

**Opt-funct.**		PBE	PBE	PBE	B3LYP	rSCAN	rSCAN	rSCAN	rSCAN
**Opt-E_{cut}**		600	900	900	600	600	600	900	900
NMR-funct.		PBE	PBE	PBE	PBE	rSCAN	PBE	rSCAN	rSCAN
NMR-E_{cut}		600	600	900	600	600	600	900	600
Atom	Exp								
H1	14.0	15.56	15.61	15.73	16.41	15.59	16.06	15.83	15.70
H2	8.8	20.19	20.21	20.34	20.85	19.78	20.26	20.27	20.13
H3	6.8	22.97	23.03	23.16	23.55	23.53	23.90	23.57	23.40
H4	6.1	24.34	24.37	24.52	24.65	24.10	24.53	24.24	24.05
H5	6.8	23.38	23.39	23.54	23.76	23.12	23.54	23.29	23.12
H6	6.8	22.71	22.72	22.87	23.29	22.54	22.98	22.75	22.57
H7		28.80	28.81	28.97	29.34	28.56	29.08	28.71	28.54
H8	0.8	29.86	29.89	30.04	30.41	29.54	30.12	29.72	29.54
H9		29.48	29.51	29.66	30.03	29.17	29.70	29.42	29.23
H19	14.7	14.02	13.98	14.08	15.20	14.01	14.21	14.39	14.26
H20	6.1	23.82	23.85	23.99	24.28	23.58	24.05	23.69	23.51
H23	6.1	23.71	23.74	23.88	24.24	23.55	23.97	23.67	23.50
	MAE	0.27	0.28	0.28	0.20	0.35	0.36	0.30	0.30
	E_{max}	0.56	0.56	0.56	0.59	0.86	0.78	0.62	0.61
	Slope	-1.094	-1.096	-1.100	-1.046	-1.077	-1.091	-1.060	-1.056
	Intercept	30.432	30.471	30.635	30.669	30.178	30.711	30.262	30.062
C1	153.8	22.13	22.15	21.95	25.21	33.65	24.26	33.52	33.75
C2	116.3	55.13	55.13	55.01	58.30	66.59	57.00	65.85	66.09
C3	144.2	27.07	27.06	26.91	31.02	39.88	28.76	39.56	39.90
C4	121.5	47.73	47.69	47.54	52.23	59.52	49.59	59.56	59.74
C5	134.7	34.91	35.05	34.88	39.67	47.92	37.49	47.76	48.11
C6	18.7	154.15	154.17	154.11	157.98	159.35	156.17	159.48	159.56
C13	166.6	1.85	2.44	1.67	9.47	16.38	5.78	15.73	16.36
C14	136.1	31.18	31.13	30.93	35.36	44.32	32.99	43.82	44.07
C17	172.7	-2.66	-2.02	-2.92	4.51	11.74	0.69	11.34	12.03
C18	136.1	31.43	31.39	31.23	35.34	44.41	33.31	43.55	43.88
	MAE	1.78	1.81	1.77	1.86	1.38	1.76	1.45	1.47
	E_{max}	5.85	5.69	5.85	4.01	4.16	5.53	4.47	4.30
	Slope	-1.016	-1.013	-1.017	-0.999	-0.957	-1.008	-0.961	-0.957
	Intercept	172.389	172.146	172.379	174.861	176.838	173.700	176.962	176.873
N1		64.30	64.49	63.03	70.87	79.80	68.69	78.69	79.88
N2		137.67	137.85	136.60	142.46	150.32	142.38	148.32	149.52

Table S9. Experimental chemical shifts (ref. [18] in the main text) and calculated shieldings (ppm) of DUTNUC optimized as a salt, as a cocrytsal (with fixed O–H distance), and weighted average of these two forms. The atom numbering corresponds to the numbering in the crystal structure deposited in CSD.

**Opt-funct.**		Salt<br>PBE	Cocrystal<br>PBE	83% Salt<br>+	Salt<br>rSCAN	Cocrystal<br>rSCAN	83% Salt<br>+
Opt-E_{cut}		600	600	17% Cocryst	600	600	17% Cocryst
NMR-funct.		PBE	PBE		rSCAN	rSCAN	
NMR-E_{cut}		600	600		600	600	
Atom	Exp						
H1	14.0	15.56	12.84	15.10	15.59	13.82	15.29
H2	8.8	20.19	22.37	20.56	19.78	21.63	20.09
H3	6.8	22.97	24.61	23.25	23.53	24.71	23.73
H4	6.1	24.34	25.03	24.46	24.10	25.00	24.25
H5	6.8	23.38	23.67	23.43	23.12	23.42	23.17
H6	6.8	22.71	23.09	22.77	22.54	22.62	22.55
H7		28.80	29.13	28.86	28.56	28.70	28.58
H8	0.8	29.86	29.94	29.87	29.54	29.58	29.55
H9		29.48	29.59	29.50	29.17	29.17	29.17
H19	14.7	14.02	17.95	14.69	14.01	16.40	14.42
H20	6.1	23.82	23.62	23.79	23.58	23.40	23.55
H23	6.1	23.71	23.44	23.66	23.55	23.15	23.48
	MAE	0.27	1.05	0.24	0.35	0.85	0.32
	E_{max}	0.56	3.17	0.57	0.86	1.88	0.75
	Slope	-1.094	-1.032	-1.083	-1.077	-1.041	-1.070
	Intercept	30.432	30.564	30.454	30.178	30.344	30.206
C1	153.8	22.13	16.58	21.19	33.65	25.49	32.26
C2	116.3	55.13	60.23	56.00	66.59	71.82	67.48
C3	144.2	27.07	30.55	27.66	39.88	43.87	40.56
C4	121.5	47.73	48.59	47.88	59.52	60.28	59.65
C5	134.7	34.91	23.57	32.98	47.92	34.11	45.57
C6	18.7	154.15	152.6	153.89	159.35	158.94	159.28
C13	166.6	1.85	3.16	2.07	16.38	17.47	16.57
C14	136.1	31.18	31.89	31.30	44.32	44.95	44.43
C17	172.7	-2.66	2.78	-1.74	11.74	18.70	12.92
C18	136.1	31.43	31.49	31.44	44.41	46.53	44.77
	MAE	1.78	3.46	2.06	1.38	4.12	1.69
	E_{max}	5.85	11.93	4.90	4.16	14.43	2.71
	Slope	-1.016	-1.001	-1.013	-0.957	-0.950	-0.956
	Intercept	172.389	170.335	172.040	176.838	175.749	176.653

Table S10. The calculated shieldings (σ) and chemical shifts (δ) of the N–H and O–H protons in DUTNUC and the corresponding bond distances (Å). All NMR calculations were performed with the PBE functional and E_{cut} = 600 eV.

		***d*(O–H)**	**σ**	**δ**	**δ_{Exp}**
H19	B3LYP	1.011	15.20	14.79	14.7
	rSCAN	1.021	14.21	15.13	
	PBE	1.034	14.02	15.00	
	PIMD[a]	1.091	12.61	15.32	
		d(N–H)	σ	δ	δ_{Exp}
H1	B3LYP	1.039	16.41	13.63	14.0
	rSCAN	1.044	16.06	13.43	
	PBE	1.055	15.56	13.59	
	PIMD[a]	1.083	14.99	13.32	

[a]All N–H and O–H distances in the PBE-optimized structure were manually adjusted to the average distances obtained from the PIMD simulation.
